# Life-threatening risk factors contribute to the development of diseases with the highest mortality through the induction of regulated necrotic cell death

**DOI:** 10.1038/s41419-025-07563-7

**Published:** 2025-04-11

**Authors:** Zsuzsa Muszka, Viktória Jenei, Rebeka Mácsik, Evgeniya Mezhonova, Silina Diyab, Réka Csősz, Attila Bácsi, Anett Mázló, Gábor Koncz

**Affiliations:** 1 Department of Immunology, Faculty of Medicine, University of Debrecen, Egyetem square 1, 4032 Debrecen, Hungary; 2 Doctoral School of Molecular Cell and Immune Biology, University of Debrecen, Egyetem square 1, 4032 Debrecen, Hungary; 3 Gyula Petrányi Doctoral School of Allergy and Clinical Immunology, University of Debrecen, Egyetem square 1, 4032 Debrecen, Hungary

**Keywords:** Necroptosis, Chronic inflammation

## Abstract

Chronic diseases affecting the cardiovascular system, diabetes mellitus, neurodegenerative diseases, and various other organ-specific conditions, involve different underlying pathological processes. However, they share common risk factors that contribute to the development and progression of these diseases, including air pollution, hypertension, obesity, high cholesterol levels, smoking and alcoholism. In this review, we aim to explore the connection between four types of diseases with different etiologies and various risk factors. We highlight that the presence of risk factors induces regulated necrotic cell death, leading to the release of damage-associated molecular patterns (DAMPs), ultimately resulting in sterile inflammation. Therefore, DAMP-mediated inflammation may be the link explaining how risk factors can lead to the development and maintenance of chronic diseases. To explore these processes, we summarize the main cell death pathways activated by the most common life-threatening risk factors, the types of released DAMPs and how these events are associated with the pathophysiology of diseases with the highest mortality.

**Figure:**
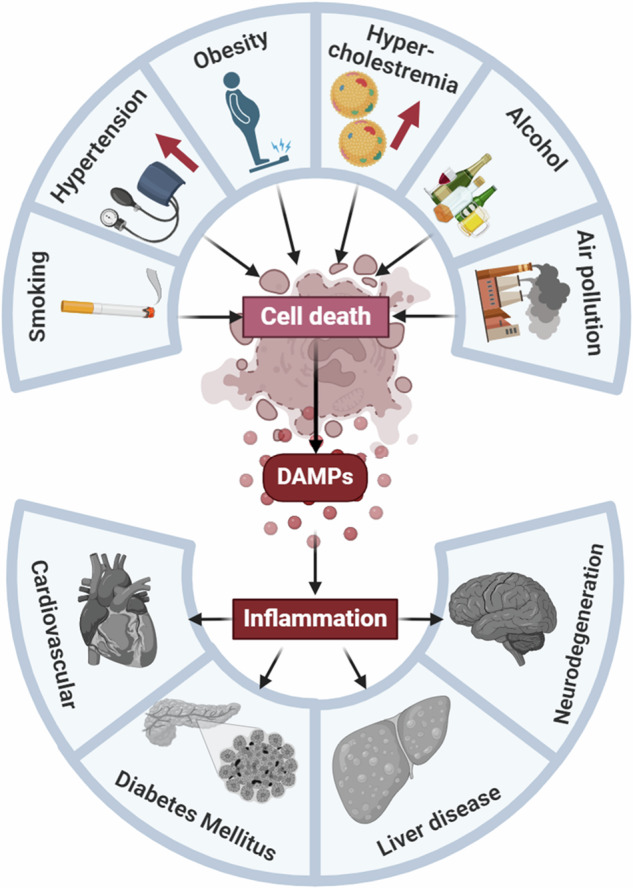
Various risk factors, such as smoking, air pollution, alcoholism, hypertension, obesity, and high cholesterol levels induce regulated necrosis. Subsequently, the release of DAMPs leads to chronic inflammation, which increases the risk of many diseases, including those with the highest mortality rates.

Various risk factors, such as smoking, air pollution, alcoholism, hypertension, obesity, and high cholesterol levels induce regulated necrosis. Subsequently, the release of DAMPs leads to chronic inflammation, which increases the risk of many diseases, including those with the highest mortality rates.

## Facts

- Environmental, physiological or behavioral risk factors can induce regulated necrotic cell death and DAMP production.
- DAMP-related sterile inflammation plays a role in the development and progression of cardiovascular diseases, neurodegenerative diseases, diabetes or alcoholic and non-alcoholic liver diseases.
- Current anti-inflammatory treatments do not target the root cause of cell death processes and the release of DAMPs.

## Open questions

- To what extent can the harmful effects of risk factors be mitigated by regulating necrotic cell death?
- To what extent do the DAMP patterns of pathologies associated with sterile inflammation overlap?
- In which diseases can drugs targeting the pathomechanism of sterile inflammation be used, such as drugs that inhibit the effects of regulated cell death or DAMPs?

## Introduction

Over 50% of global deaths are associated with a fairly limited spectrum of conditions, including cardiovascular and neurodegenerative diseases, diabetes mellitus, autoimmune disorders, as well as various organ-specific diseases and certain types of cancers. Extensive research conducted over the past few decades has demonstrated that the pathogenesis of each of these disorders is tightly associated with sterile inflammation [1].

The inflammatory response is primarily triggered by cells of innate immunity upon activation of pattern recognition receptors (PRRs). PRRs detect both pathogen-associated molecular patterns (PAMPs) and damage-associated molecular patterns (DAMPs). Accordingly, sterile inflammation, in the absence of pathogens, is predominantly triggered by DAMPs released by cell or tissue damage. Sterile inflammation is a common phenomenon that occurs in various contexts, including autoimmune and autoinflammatory disorders, as a hallmark feature of tumors, in response to sports injuries, and as a consequence of certain genetic deficiencies. During the resulting inflammation, the activation of effector cells of the immune system leads to tissue degeneration, which can be compensated by the subsequent tissue regeneration steps [2].

The release of DAMPs typically occurs when cells undergo necrosis, and consequent membrane rupture leads to the leakage of intracellular components, generating danger signals [3]. In addition to the primary necrosis, several regulated necrotic cell death modalities have been identified, such as necroptosis, pyroptosis, ferroptosis and parthanathos. Although different forms of cell death can be triggered by a wide spectrum of stimuli, all are characterized by a loss of membrane integrity and the consequent necroinflammation [4].

Risk factors, including environmental (air pollution), physiological (high blood pressure, obesity, high blood cholesterol), and even behavioral risk factors (smoking, alcoholism), are all closely related to life-threatening disorders. It is not clear how each of the different lifestyle risk factors affects diseases with such different pathomechanisms, but a common characteristic of all these factors is that they initiate sterile inflammation. The effects of different risk factors can converge in necrotic cell death, which leads to DAMP release and then inflammation. Since inflammation is not only the consequence of cell death, but can also its cause through tissue destruction, the process can turn into a chronic reaction.

While the impact of health hazards on the most lethal diseases is well documented, how these factors influence necrotic cell death and DAMP production is poorly understood. We first summarize our knowledge of how different life-threatening risk factors influence the necrotic cell death pathways and then review how regulated necrotic cell death and DAMP-related chronic inflammation can lead to the development of high-mortality diseases.

Although cancer is also one of the leading causes of death, and about 25% of cancers are related to chronic inflammation [5], we do not cover this disease in our article. Specific processes characteristic of the pathomechanism of tumors, such as mutagenesis, abnormal proliferation, and metastasis, make it difficult to compare cancer with the other diseases mentioned. Comprehensive reviews of the complex roles of chronic inflammation, cell death and DAMPs in cancer processes are available to readers [6–9].

## Cell death pathways and DAMP release in chronic inflammation

**Fig. 1:**
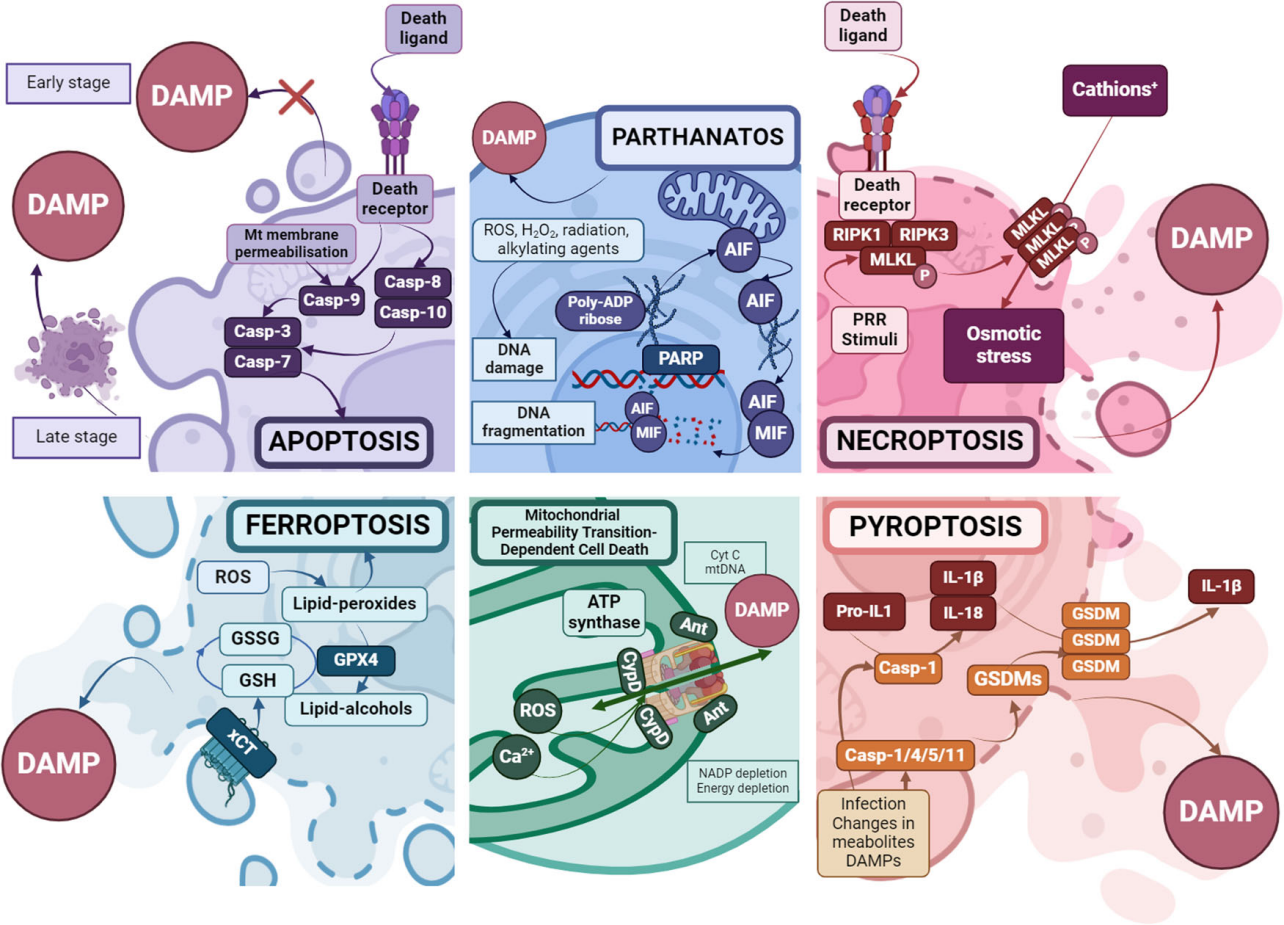
Schema of signaling pathways of cell death. Apoptosis is carried out by regulated mechanisms triggered by various stress signals through the intrinsic pathway, and death receptors via the extrinsic pathway. In the intrinsic pathway, cytochrome-C is released from the mitochondrial intermembrane space, leading to the activation of caspase-9. In the extrinsic pathway, ligand binding to death receptors triggers the activation of caspase-8 and -10. Initiator caspases (caspases-9, -8, and -10) activate effector caspases, such as caspases-3 and -7, which carry out the controlled, energy-dependent dismantling of the cell. Apoptosis is characterized by cell shrinkage and membrane blebbing, followed by the formation of apoptotic bodies. These apoptotic bodies are typically engulfed by efferocytes or neighboring cells, preventing the release of damage-associated molecular patterns (DAMPs) and minimizing inflammation. Necroptosis is a form of regulated necrosis, which is activated by the necrosome containing Receptor-interacting protein kinase 1 (RIPK1) and RIPK3 (after stimulation of death receptors). Necrosome activates mixed lineage kinase domain-like protein (MLKL) by phosphorylation, which results in its oligomerization and translocation to the plasma membrane. MLKL pores compromise membrane integrity, allowing an increased influx of cations, leading to osmotic imbalance. This imbalance causes cell swelling, membrane rupture, and the subsequent release of DAMPs into the extracellular space. During pyroptosis, caspases—primarily caspase-1, which is activated by NOD-like receptors (NLR) organized inflammasomes, or by caspases-4/5 (homologous to caspase-11 in mice), which are activated by lipopolysaccharide (LPS)—cleave gasdermin proteins to initiate cell death. The ASC adapter protein also promotes the activation of caspase-1 upon various stimuli. After the cleavage of gasdermin proteins their N-terminal domains oligomerize and create pores in the plasma membrane leading to cell death. These pores are not ion-selective, thus do not lead to osmotic shock. Additionally, activated caspase-1 cleaves the pro-forms of the inflammatory cytokines IL-1β and IL-18 into their mature forms, facilitating their release. Ferroptosis is a regulated cell death induced by iron-dependent lipid peroxidation. It can occur due to increased ROS levels (which may be a consequence of iron-mediated Fenton reactions) or a reduced capacity of the cell’s antioxidant system. The oxidation of phospholipids containing polyunsaturated fatty acids increases lipid peroxide levels, leading to enhanced membrane permeability and potential rupture. The oxidative balance of the cells is maintained by the cooperation of the xCT transporter and glutathione peroxidase 4 (GPx4). xCT ensures the transport of cysteine, a glutathione precursor, into the cell, while GPx4 protects cells from oxidative stress by catalyzing the reduction of lipids and other organic hydroperoxides to their corresponding alcohol forms, using glutathione as a substrate. Parthanatos is initiated by hyperactivation of PARP1, a key enzyme in the DNA damage response, leading to depletion of the PARP substrates NAD+ and ATP. As a result, the cells’ energy source is exhausted. In addition to cellular energy deficiency, cell death is accompanied by the release of apoptosis-inducing factor (AIF) from the mitochondria, which is translocated to the nucleus after parylation by the PARP enzyme and induces Macrophage migration inhibitory factor (MIF)-dependent DNA fragmentation. Activation of mitochondrial permeability transition (MPT) is a consequence of intracellular stress signals that lead to dysregulation of the inner mitochondrial membrane. This can cause osmotic rupture of both mitochondrial membranes, resulting in cyclophilin D (CYPD)-dependent necrosis. CYPD in conjunction with a voltage dependent anion channel (VDAC) and adenine-nucleotide translocase (ANT) forms the permeability transition pore complex. Under oxidative stress, low ATP levels, or high Ca2+ concentrations, this complex forms open pores between the inner and outer mitochondrial membranes. This results in mitochondrial swelling and allows unregulated diffusion of molecules, ions and apoptogenic proteins. For a deeper understanding of signaling pathways, we recommend the review of the Cell Death Nomenclature Committee [4]. Apoptosis-associated speck-like protein containing a C-terminal caspase recruitment domain (ASC), Apoptosis-inducing factor mitochondria associated 1 (AIF), Cyclophilin D (CyPD), cystine/glutamate antiporter (xCT), damage–associated molecular pattern (DAMP), Gasdermin (GSDM), glutathione GSH, glutathione peroxidase (GPx4), inner mitochondrial membrane (IMM), Macrophage migration inhibitory factor (MIF), mitochondrial permeability transition (MPT), Mixed lineage kinase domain-like protein (MLKL), NOD-like receptor (NLR), oxidized glutathione (*GSSG*), pattern recognition receptor (PRR), Poly(ADP-ribose) polymerase 1 (PARP-1), Receptor-interacting serine/threonine-protein kinase 1 (RIPK1), reactive oxygen species (ROS).

**Fig. 2:**
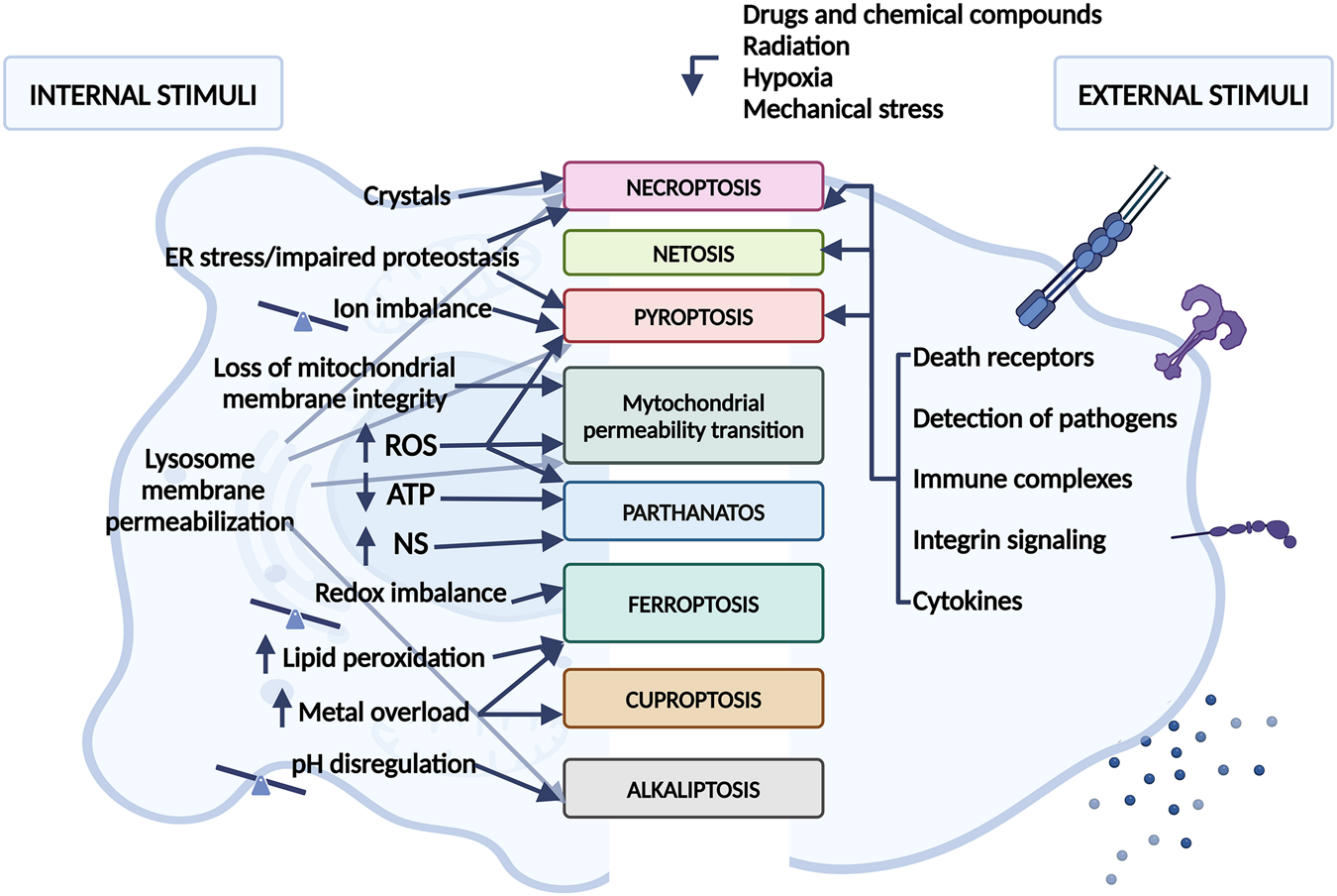
Various stimuli trigger regulated cell death processes.

In recent decades, several regulated necrotic cell death modalities have been described (Fig. 1). During the immune response, activation of cytotoxic cells through death receptors, detection of pathogens via pattern recognition receptors, integrin signaling, immune complexes, or various cytokines can all stimulate necroptosis, pyroptosis, and netosis [4]. In addition to immune system-related stimuli, many extra- or intracellular disturbances can trigger regulated necrosis (Fig. 2), such as ion imbalance (pyroptosis), pH dysregulation (alkaliptosis), ATP depletion (parthanathos), generation of reactive oxygen species (parthanathos, pyroptosis, mitochondrial permeability transition), redox imbalance (ferroptosis), metals overload (ferroptosis, cuproptosis), lipid peroxidation (ferroptosis), nitrogen species formation (parthanatos), hypoxia (parthanatos) and exposure to various drugs, chemical compounds or radiation [4, 10].

Under sterile conditions, tissue or cellular damage leads to various forms of necrotic cell death, which trigger DAMP-mediated inflammation. DAMPs are endogenous molecules that are not recognized by the immune system under normal physiological conditions. For example, DAMPs characteristic of disrupted extracellular matrix include versican, biglycan, decorin, while intracellular components released from necrotic cells can include ATP, HMGB1, histons, nuclear DNA, mitochondrial factors (such as N-formyl peptides, mtDNA), lysosomal proteins (e.g., Cold-inducible RNA-binding protein), and endoplasmic reticulum stress signals (such as PKR-like endoplasmic reticulum kinase or calreticulin), all of which can act as alarm signals [11].

Particular regulated necrotic cell death processes may differ in their molecular background, pore-forming proteins, and the pattern of released molecules [10]. NINJ1-mediated membrane disruption is characteristic of the late stages of many necrotic processes [12]. Therefore, DAMP release may differ in individual cell death processes, and possibly even in their phases.

In addition, DAMPs can act synergistically with PAMPs, but they are also responsible for the immune response independent of infection. As a result of tissue or cell damage, DAMPs activate the immune response, especially the components of innate immunity, alerting it to unwanted pathological reactions [13]. They can stimulate the production of chemokines to attract the cells of the immune system to the site of danger and facilitate the production of inflammatory cytokines [14]. The inflammatory response mediated by DAMPs ultimately translates into subsequent tissue regeneration [11, 15].

The role of individual DAMPs in sterile inflammation has been investigated in various in vivo models. Administration of recombinant DAMPs leads to sterile inflammation and some human pathologies are directly linked to elevated DAMP levels. Recombinant IL-1α, HMGB1 and S100A9 exhibited immune adjuvant activity, increased the overall incidence of arthritis and induced an inflammatory response in the lung in mouse models, respectively [16–18]. Overproduction of IL-1β results in systemic inflammatory diseases, and elevated serum uric acid leads to gout and nephrolithiasis in humans [19, 20].

Neutralization or inactivation of specific DAMPs may be beneficial in several inflammatory diseases. Anti-HMGB1 was shown to be protective in arthritis models [21], and in high-fat diet-induced atherosclerosis in ApoE-/- mice [22]. Canakinumab, a monoclonal antibody that targets IL-1β, reduced inflammatory markers and cardiovascular events in patients with prior myocardial infarction [23]. Neutralization of extracellular ATP with apyrase reduced systemic injury in an experimental acute pancreatitis model [24]. Administration of RNase1 significantly reduced the incidence of myocardial infarction [25], while DNase-1 treatment reduced the inflammatory response in a rat ischemia-reperfusion model [26].

Release of DAMPs exacerbates various inflammatory processes, and persistent activation of cell death pathways can lead to chronic inflammation caused by DAMP production unless the resolution mediators become dominant [11, 27]. This process is also involved in the development of diseases such as cardiovascular diseases, neurodegenerative disorders, diabetes or alcoholic and non-alcoholic liver diseases (Table 1).

**Table 1:** Risk-factor-induced cell death processes and DAMP secretion.

Environmental factor	Cell death	In vivo evidences	DAMPs
Air pollution	Apoptosis [198]		uric acid [199] S100 [200] HSP [201] ATP [37] HMGB1 [39] mtDNA [202] ROS [203] biglycan [204]
	Necroptosis [205]		
	Pyroptosis [33]	Nlrp3 KO mice were resistant to diesel exhaust particles-induced neurotoxicity [206].	
	Ferroptosis [34]	Nrf2 deficiency reduced inflammation in liver and adipose tissue in response to PM2.5 exposure [207].	
High blood pressure	Apoptosis [46]		HMGB1 [208] DNA mtDNA [209] ROS [210] Uric acid [211] S100 [56] Decorin [212] IL-33 [213]
	Necroptosis [47]		
	Pyroptosis [214]	*Blood pressure* was not different in wild-type or c*aspase*-1-deficient *mice* [215]. Gsdmd−/− mice exhibited less vascular damage in Ang II osmotic mini-pumps induced hypertensive vascular remodeling [216]. ASC KO, but not NLRP3 KO shows attenuated pulmonary hypertension and reduced levels of inflammatory cytokines [217].	
	Ferroptosis [218]	Systolic and diastolic blood pressure were decreased in Nrf2 KO mice [219].	
Obesity	Apoptosis [220]		HMGB1 [77] IL-1β [221] mtDNA [222] DNA [223] RNA [224] Calreticulin [225] ROS [226] S100 [227] Biglycan [228] Decorin [229] Versican [230] LMW hyaluronan [231] IL-33 [232] [233]
	Necroptosis [65]	RIPK3 and MLKL deficiency reduces inflammation and glucose intolerance in different obesity models [234].	
	Pyroptosis [234]	NLRP3 or GSDMD deficiency reduces adipose tissue inflammation [234].	
	Ferroptosis [235]	Nrf2 KO mice have an improved obesity phenotype [236].	
Cholesterol levels	Apoptosis [186]		HMGB1 [93] ROS [91] Decorin [237]
	Necroptosis [83]	RIPK3 gene deficiency delayed mortality in mouse models of cholesterol metabolism dysfunction [83]. In high-fat, high-fructose, and high-cholesterol diet models, Mlkl-/- mice were protected from liver damage [238].	
	Pyroptosis [239]	Deletion or pharmacological inhibition of NLRP3 did not protect against liver injury in high-fat diet models [240].	
	Ferroptosis [90]	High-cholesterol diet-induced upregulation of gene expression related to lipid and bile acid metabolism was not induced in Nrf2-null mice [241].	
Smoking	Apoptosis [242]		HMGB1 [243] IL-1β [99] DNA [106] mtDNA ROS [244] S100 [245] Biglycan [246] LMW hyaluronan [247] galectins [248] LL-37 [248] a decrease in IL-33 [249]
	Necroptosis [106]	Deletion of Ripk3 or Mlkl protected against CS-induced airway inflammation [95].	
	Pyroptosis [250]	Nlrp3 and caspase-1 deficiency are not critically implicated in CS-induced inflammation, but IL-1R KO mice were protected against CS exposure-induced pulmonary inflammation [99].	
	Ferroptosis [102]	Infiltration of immune cells, and inflammatory cytokine production increased in bronchoalveolar lavage fluid in CS-exposed GPX4+/− mice [102]. Genetic ablation of Nrf2 enhances susceptibility to CS–induced emphysema in mice [103].	
	Parthanatos [251]		
Intensive alcohol consumption	Apoptosis [252]	Ablation of Caspase-8 does not prevent alcoholic liver injury in mice [253].	HMGB1 [249] IL-1β [254] mtDNA [122] mtdsRNA [122] miRNA [122] ATP [122] ROS [126] Uric acid
	Necroptosis [255]	Ablation of RIPK3 can effectively prevent alcohol-induced liver damage [172]. *Mlkl*–/– does not completely protected from Gao-binge– or chronic ethanol-induced liver injury [256].	
	Pyroptosis [257]	Deficiency of NLRP3, Casp-1, ASC, IL-1R1, and caspase-11 prevented alcohol-induced inflammation and liver damage [180], [119], [173].	

## Risk factor-induced cell death processes and DAMP secretion

It is well known that environmental, physiological and behavioral risk factors such as air pollution, high blood pressure, obesity, cholesterol levels, smoking or intensive alcohol consumption significantly contribute to the increased risk of cardiovascular and neurodegenerative diseases, diabetes and various organ-specific disorders. These life hazards are known to directly contribute to the exacerbation of disorders by inducing cell death and DAMP secretion leading to chronic inflammation, as detailed in the subsequent sections and Table 1.

## Air pollution-related cell death processes and DAMP secretion

Particulate matter (PM) and/or adsorbed chemicals are risk factors for the development of pulmonary diseases and have become a major concern worldwide. Air pollution results in endoplasmic reticulum stress and mitochondrial dysfunction, promoting pancreatic β-cell apoptosis, which impairs insulin synthesis over the long-term [28]. Necroptosis can also be induced by heavy metals, as CdCl2 exposure has been shown to induce necroptosis and hepatic injury both in vivo and in vitro [29]. Exposure of A549 epithelial or RAW264.7 macrophage cell lines to PM with an aerodynamic diameter of less than 2.5 μm (PM2.5) or black carbon, a key component of PM2.5, caused necroptosis and consequently enhanced inflammation [30, 31]. Necroptosis also contributes to PM exposure-related inflammation during ocular surface injuries [32].

Various factors of environmental pollution, like water-soluble inorganic ions, carbonaceous materials, and heavy metals in PM2.5, activate the NLRP3 inflammasome and pyroptosis. Inflammatory responses associated with elevated levels of IL-1β in bronchoalveolar lavage fluid (BALF) were shown in mice exposed to PM2.5. Wood smoke particulate matter activates the NLRP3 inflammasome by regulating extracellular ATP levels and mediates pyroptosis in human bronchial epithelial 16-HBE cells [33]. PM2.5 significantly increased mitochondrial dysfunction, causing oxidative damage and GPX-dependent ferroptotic cell death in neurons [34]. Additionally, PM2.5 enhances the ferroptosis sensitivity of HUVEC cells, inducing iron overload, lipid peroxidation, GSH depletion, and redox imbalance [35]. Heavy metal components of air pollution, such as As, Pb, and Cd can also induce redox imbalance causing ferroptosis in the liver, central nervous system, and the kidneys [36].

Inhaling air pollutants disrupts the epithelial barrier, causing oxidative stress [37]. PM2.5 directly activates NLRP3 by various substances, such as elevated extracellular ATP [38]. Similarly, PM2.5-induced ROS production and systemic oxidative stress increase DAMP production, further activating the NLRP3 inflammasome and IL-1 family cytokine production [37]. Consequently, elevated IL-1β and IL-33 expression regulate airway barrier function [39]. PM2.5-induced cell death causes the secretion of HMGB1, mtDNA, and N-formyl peptides triggering inflammation in the airways of mice [39, 40]. In motor vehicle extract-treated rats, a larger ratio of HMGB1 and RAGE-positive bronchial epithelial cells were detected in the lungs and in vitro in the supernatant of a human bronchial epithelial cell line [41]. Furthermore, organic dust was found to cause sustained neuroinflammation in in vivo mice study involving HMGB1 and RAGE signaling [42].

## Blood pressure-related cell death processes and DAMP secretion

Hypertensive stimuli such as altered mechanical forces, increased velocity of blood flow and friction lead to endothelial dysfunction and induce multiple modes of vascular cell death [43–45]. An increase in blood pressure leads to ER stress resulting in apoptosis of cardiomyocytes with increased caspase-3 expression in a rat model [46]. RIPK3-mediated necroptosis and its role in the progression of inflammation have also been identified in the pathogenesis of pulmonary arterial hypertension (PAH) [47, 48].

Both canonical and non-canonical pyroptosis pathways have been implicated in hypertension. Caspase-11 deficiency in vivo and caspase-4 silencing in the human pulmonary arterial endothelial cells in vitro alleviated the development of PAH [49], and inhibition of Caspase-1 also attenuated the pathogenesis of PAH [50].

The role of ferroptosis in hypertension is controversial. PAH is characterized by decreased serum iron levels. However, whether iron deficiency contributes to the disease or is merely a consequence of it remains debated [51]. Solute carrier family member 7, 11 (SLC7A11), the Xc subunit of the cystine/glutamate antiporter system which inhibits ferroptosis, is upregulated in PAH patients and hypoxia-induced PAH rat models [52]. On the contrary, ferroptosis was observed in pulmonary artery endothelial cells from a monocrotaline-induced PH in a rat model and was characterized by the upregulation of the labile iron pool and lipid peroxidation, and the downregulation of GPX4 expression.

It’s suggested that, high blood pressure and pro-hypertensive factors induce vascular injury. In patients with idiopathic or congenital heart disease-related PAH, HMGB1 release is elevated [53, 54]. Mitochondrial dysfunction also plays a role in PAH, activating inflammasomes [53, 55]. Inflammasome activation results in the production of interleukin IL-1β and IL-18, both of which are key biomarkers of PAH. Accordingly, clinical trials using IL-1β blockade have shown reductions in recurrent cardiac events and inflammation [53]. Moreover, increased serum levels of S100B are detected in hypertension [56], and S100a8/a9 produced by neutrophils was identified as an initial proinflammatory factor during acute hypertension [57]. Hypertension and mechanical stress contribute to endothelial injury which further accelerates the development of atherosclerosis via the activation of oxidative stress, leading to lipid oxidation and tissue damage [58–60]. Markedly, one of the most common risk factors for brain aging is arterial hypertension, causing endothelial damage that disrupts the blood-brain barrier, and is associated with the production of ROS and β-amyloid, which causes neuroinflammation [61].

## Obesity-related cell death processes and DAMP secretion

Obesity causes low-grade chronic inflammation due to the release of adipokines and cytokines from fat cells. Also, the destruction of fat cells is a major factor triggering the release of inflammatory substances, attracting macrophages around the necrotizing cells [62].

Both the intensity of the death receptor- and mitochondria-mediated apoptotic pathways increased in adipocytes from humans and mice with obesity and insulin resistance [63], but the role of necrotic cell death in obesity has also been intensively investigated. Functionally, increased RIPK1 expression in adipose tissue has been proven, however, the causative role of necroptosis in obesity remains unclear [64, 65].

The NLRP3 inflammasome contributes to obesity-induced inflammation, and ablation of NLRP3 in mice prevents obesity-induced inflammasome activation in fat depots [66]. Caspase-1 protein levels are more abundantly expressed in the adipose tissue of hyperglycemic ob/ob animals and high glucose levels activate Caspase-1 in human and murine adipose tissue [67]. Nonetheless, GSDMD deficiency does not protect mice against HFD-induced adipose tissue inflammation [68]. AGEs contribute to the acceleration of the development of atherosclerosis through activation of the NLRP3-ASC inflammasome pathway in a mouse monocyte cell line, and by increasing the level of HMOX1 (a member of the ferroptotic pathway) in primary human aortic endothelial cells [69–71]. Ferroptosis is intricately linked to obesity [72], and it is characterized by iron buildup, lipid peroxide hyperplasia, GPX4 inhibition, and systemic Xc inhibition in the adipose tissue under obesity conditions [73]. Several studies have shown that overexpression of GPX4 is protective against adipocyte inflammation, while iron chelators reduce the generation of inflammatory agents and the infiltration of adipose tissue macrophages [74].

In obesity, apoptotic, necroptotic, and pyroptotic adipocytes are the major sources of DAMPs in visceral fat tissue [75]. The mechanically stressed adipocytes secrete factors, like HMGB1 that attract immune cells, and favor M1-like macrophage differentiation. HMGB1 levels are increased in adipose tissue in obese individuals compared to those of normal-weight [76]. Moreover, it was shown that the deficiency of its receptor, TLR4, decreases inflammation and insulin resistance in adipose tissue. Furthermore, adipocyte death raises free fatty acid levels, causing mitochondrial dysfunction, mtDNA release, ROS accumulation, and inflammasome activation in endothelial cells [77, 78]. Additionally, DNA released by white adipose tissue can enhance inflammation together with secreted HMGB1 [77]. As well as, hyperglycemia causes ROS production, which promotes the development and progression of diabetes through the induction of M1-like pro-inflammatory macrophages [79].

## High cholesterol level-related cell death processes and DAMP secretion

Cholesterol accumulation in macrophages and other immune cells mediates cell death, both apoptotic and necrotic processes, and promotes inflammatory responses as well [80, 81]. In addition to cholesterol-induced intrinsic [82], and extrinsic apoptosis [81], necroptotic death also plays a role in the development of atherosclerosis. While elevated levels of phosphorylated RIPK3 were detected in atherosclerotic plaques, knockdown of RIPK3 showed delayed mortality compared to apolipoprotein E (ApoE) single knockout mice [83]. Moreover, ox-LDL and cholesterol crystals induce NLRP3 inflammasome activation, upregulate the expression of various necroptotic molecules (RIPK3, MLKL), and activate ferroptotic (HMOX-1) protein [84–87].

The cholesterol accumulation disrupts lysosomal membrane structure leading to cathepsin B release, which activates NLRP3 and induces pyroptosis [88]. Furthermore, cholesterol crystals, together with the complement system, also induce NLRP3 activation [89]. However, cholesterol directly enhances ferroptosis resistance in hematopoietic stem cells [90].

Additionally, extracellular free cholesterol was demonstrated to enhance ROS production [91], and oxDNA levels are significantly increased in the plasma of obese individuals with NASH [78]. In HFD-fed obese mice, obesity induced blood-brain barrier damage, microglial activation, elevated proinflammatory cytokines, and increased oxidative stress [92]. Another risk factor for vascular endothelium damage is ox-LDL which upon its accumulation results in the secretion of DAMPs. Accordingly, ox-LDL upregulates HMGB1 expression in human umbilical vein endothelial cells [93].

## Smoking-related cell death processes and DAMP secretion

Smoking is one of the most significant factors that increases the risk of many diseases and contributes to life-threatening pathological processes. Cigarette smoke (CS) contains thousands of chemicals, several of which can have cytotoxic effects on human tissues and organs.

It has long been acknowledged that CS induces apoptosis of bronchial epithelial cells, but it has now been proven that it can also induce necrotic cell death in these cells [94]. RIPK3 or MLKL-deficient mice showed reduced airway inflammation in a chronic CS-induced COPD model [95] and expression of necroptosis-associated markers (RIPK1, RIPK3, and MLKL) were elevated in cigarette tar treated vascular smooth muscle cells [96].

Furthermore, CS stimulates NLRP3 inflammasome-induced pyroptosis and subsequent increase in IL-1β and IL-18 levels in human and mouse models [97, 98]. CS-induced acute pulmonary inflammation was not affected by NLRP3 deficiency but was attenuated upon IL-1α or IL-1β neutralization [99]. It was established that, compared to non-smokers, heavy smokers with coronary artery disease display activation of the NLRP3 inflammasome which facilitates the progression of atherosclerosis [100].

Ferroptosis has also been shown to play a role in cigarette smoke-induced atherosclerosis [101]. In addition, CS induces ferroptosis in human bronchial epithelial cells in culture, a process that is attenuated by the treatment with an iron chelator or ferroptosis inhibitors. Consistent with its role in ferroptosis, mice overexpressing GPX4 have a milder response to CS [102]. Additionally, the disruption of Nrf2 in mice, a transcription factor that is involved in the regulation of many antioxidant genes, leads to a widespread CS-induced emphysema than in wild-type littermates [103].

Cigarette smoking also causes DNA damage, which leads to the activation of enzymes, and PARP-1-mediated DNA repair to reduce CS-induced cell death [104]. However, overactivation of PARP-1 can lead to parthanatos cell death. CS induces the mitochondrial translocation of AIF and Endonuclease G, characteristics of parthanatos. Accordingly, the application of a specific PARP-1 inhibitor was shown to abolish the smoke-induced parthanatos pathway [105].

CS containing a complex array of toxic chemicals induces different forms of necrotic cell death in bronchial epithelial cells, consequently leading to a significant increase in DAMP levels (dsDNA, HMGB1, HSP70, mtDNA, and IL-1β) [106]. Elevated levels of HMGB1 and its receptors, RAGE and TLR4 proteins, were found in the lung tissue of smoking COPD patients [107], and CS-induced HMGB1 secretion may lead to insulin resistance [108]. Supporting this, RAGE KO mice are protected from smoke-induced lung pathologies due to reduced macrophage infiltration and chemokine release [109]. Elevated HMGB1 expression may synergize with the function of additional DAMPs, as HMGB1 forms a complex with mtDNA, leading to the co-signaling of RAGE and TLR9 receptors [110]. Moreover, RAGE activation may cause sustained ER stress and consequently the release of an ER-related DAMP, calreticulin (CRT). During the CS-induced ER stress, CRT acts as an “eat me” signal for surrounding phagocyte [110]. Furthermore, the upregulation of HMGB1 was positively correlated with IL-1β [110]. Significantly increased IL-1α levels were also detected in smokers [99]. CS triggers HSP70 production [106] activating TLR2 and TLR4 signaling and the release of proinflammatory cytokines [110]. Additionally, higher ATP levels [111], and upregulation of ATP receptors (P2X7 and P2Y2) are also observed in neutrophils, macrophages, and lung tissue of smokers. Knockout mice lacking P2Y2 receptors showed reduced lung inflammation after acute CS exposure compared to wild-type mice [112].

## Alcohol consumption-related cell death processes and DAMP secretion

Chronic alcohol exposure induces multiple forms of cellular stress, including oxidative stress, hypoxia, failed protein folding, and ER stress, resulting in the activation of both intrinsic and extrinsic pathways of apoptosis [113–115]. However, inhibition of apoptosis is not a protective mechanism in mouse models of ALD, suggesting that necroptosis may play an important role in ethanol-induced liver injury. Indeed, RIPK3 expression in the liver is elevated in both mouse and human ALD models. Additionally, RIPK3 KO mice were protected from ethanol-induced hepatocyte injury and inflammation [116].

ER stress stimulates NLRP3 activation in human steatohepatitis liver [117], and NLRP3 deficiency leads to attenuation of alcoholic steatosis [118]. Caspase-1-dependent upregulation of IL-1β also plays an important role in ALD pathogenesis according to the KO mouse model [119]. Accumulating evidence suggests the role of ferroptosis in liver diseases, including ALD. Approximately 50% of patients with alcoholic liver disease are characterized by iron overload in the liver, which contributes to liver inflammation [120]. Long-term ethanol feeding in mice was found to enhance polyunsaturated lipid peroxidation and indirectly deactivate GPX4 through severe GSH exhaustion [121].

In late-stage alcohol metabolism, diverse DAMPs (HMGB1, mtDNA, miRNA, and ATP) are secreted not only in the liver but also in adipose tissue, the gut, and bone marrow, resulting in a heightened production of proinflammatory cytokines and chemokines [122]. DAMPs amplify liver injury through the stimulation of the inflammasome [123], consequently, pro-IL-1β mRNA levels were shown to be increased in the peripheral monocytes as well as in the cerebellum of alcoholics [124]. Alcohol-associated neurodegenerative diseases have been linked to increased apoptosis, excitotoxicity, lipid peroxidation, and elevated DAMP secretion such as IL1β, ROS, mtDNA, and HMGB1 [125]. Interestingly, HMGB1 and IL-1β heterocomplexes could be detected in the postmortem human alcoholic hippocampus. Ethanol intake promotes ROS generation and raises serum uric acid levels by boosting urate production and excretion, as well as its release from dying cells [126, 127]. Additionally, serum uric acid triggers the production of inflammasome-related molecules in bone marrow-derived macrophages [128].

## The role of cell death and related DAMP release in cardiovascular diseases

Ischemic heart disease (IHD) is the leading cause of death worldwide, representing 32% of all global deaths. Of these deaths, 45% are due to myocardial infarction (MI). The main cause of IHD is the formation of atherosclerotic plaques in coronary arteries. As the disease progresses, destabilization and rupture of atherosclerotic plaques followed by thrombotic events result in complete occlusion of the coronary arteries, leading to MI. Interestingly, the outcomes of MI worsen upon restoration of the tissue perfusion, known as ischemia-reperfusion injury (IRI).

Recent studies have demonstrated the significant contribution of various cell death pathways in the progression of atherosclerosis and IHD (Table 2). Pyroptosis of endothelial cells, macrophages, and smooth muscle cells has been established to be intimately connected to the development and destabilization of atherosclerotic lesions. A significant decrease in the atherosclerotic lesion size was observed in high-fat diet mice reconstituted with NLRP3-, ASC-, IL-1α-knockout bone marrow cells [8, 88, 129, 130].

**Table 2:** Cell death and DAMP production in cardiovascular diseases.

Disease	Risk factor	Cell Death	Cell death KO mice	DAMP	DAMP KO mice
**Ischemic heart disease and ischemia-reperfusion injury**	Smoking [258] Hypertension [258] Obesity [259] Hypercholesterolemia [260] Alcohol [261] Air pollution [262]	Apoptosis [263] Necroptosis [133] Pyroptosis [190] Ferroptosis [132] Mitochondrial Permeability Transition [131]	Bax knockout tolerated heart injury upon IRI [264]. Ripk3 depletion provided cardiac protection in cardiac IR injury [265]. Nrf2 KO mice have increased infarct size and reduced cardio protection upon regional IR [266].	HMGB1 HSP60, HSP70 Fibronectin, hyaluronic acid mtDNA circulating RNA cardiac myosin extracellular ATP [134]	**-**
**Heart failure**	Smoking [267] Hypertension [268] Obesity [269] Hypercholesterolemia [270] Alcohol [271] Air pollution [272]	Apoptosis [273] Necroptosis [133] Pyroptosis [274] Ferroptosis [275] Mitochondrial Permeability Transition [131]	Bax KO mice are protected against doxorubicin-induced cardiomyopathy [276]. MLKL knockout improved myocardial function in diabetic cardiomyiopathy [277]. Inflammation, infarct development, myocardial dysfunction were diminished in ASC and Caspase-1 deficient mice [278]. GSDMD KO mice have reduced infarct size, improved cardiac function [279].	HMGB1 HSP60, HSP70 Fibronectin hyaluronic acid mtDNA circulating RNA cardiac myosin extracellular ATP [134]	**-**

Mitochondrial permeability transition pore (MPT)-mediated necrosis has also been demonstrated to have a role in the progression of atherosclerosis since the deletion of CyPD decreased the necrotic core size in advanced atherosclerotic lesions [131].

Ferroptosis may also contribute to plaque instability. GPX4 counteracts ferroptosis and inhibits the development of atherosclerosis by reducing lipid peroxidation and vascular cell sensitivity to oxidized lipids. Accordingly, GPX4 is downregulated in advanced atherosclerotic plaques in human arteries [132].

RIPK3 knockout attenuated cardiac dysfunction 30 days after MI injury [133], and necroptosis has also been demonstrated to play a significant role in cardiac IRI, since inhibition of necroptosis or global deletion of RIPK3 was shown to reduce infarct size following IRI in mice [133].

Additional studies have confirmed a strong association of NLRP3 inflammasome and pyroptosis with the pathological processes of MI and myocardial ischemia‒reperfusion injury (MIRI). Cardiac dysfunctions were significantly alleviated in ASC- or caspase-1-deficient mice, or following silencing of either NLRP3 or P2X7, a pyroptosis stimulating ATP receptor [88, 129, 130].

Furthermore, iron excess contributed to ferroptosis in cardiac cells in the early and middle stages of MI [132], and downregulation of GPX4 with subsequent ferroptosis was revealed in an MI mouse model. Following an MI, mice lacking CyPD were shown to have a smaller infarct size, as well as less adverse cardiac dysfunction and remodeling [131]. Calcium overload and oxidative stress, observed in reperfusion injury, caused MPT-induced necrosis, and inhibitors or genetic ablation of CyPD provide a strong protection from reperfusion injury [131].

Several DAMPs have been linked to the inflammatory processes underlying the progression of atherosclerosis, MI, and IRI. These include oxidized LDL (ox-LDL), cholesterol crystals and calcium phosphate crystals in the former case, as well as HSPs, HMGB1, ATP, nuclear and mtDNA, and RNA in the latter one [134]. Cholesterol crystals, ox-LDL, and calcium phosphate crystals have been shown to exert their proinflammatory effects via NLRP3 inflammasome-mediated pathways and elevated pro-IL-1 expression. In addition, excessive cholesterol crystals can promote lysosomal destabilization and rupture, resulting in the leakage of cathepsin B, further activating the NLRP3 inflammasome [135]. HSP70 is a biomarker of the clinical outcome of MIRI; however, the causal role of HSP70 in the inflammatory response after MI has not yet been proven [134]. Upon IRI, both HMGB-1 and mtDNA are released into the bloodstream. Treatment of either HMGB-1 or mtDNA infusion separately did not result in increasing infarct size, yet the combined treatment of HMGB-1 and mtDNA did have clear harmful effects [134]. It was further demonstrated that the treatment with RNase attenuates necrosis-induced cytokine production in cardiomyocytes and protects mice against IRI, marked by smaller infarct size [134].

## The role of cell death and related DAMP release in neurodegeneration

Neurodegenerative diseases are a group of disorders characterized by progressive dysfunction and loss of neurons in the central nervous system (CNS). According to the World Health Organization (WHO), currently more than 55 million people worldwide have dementia, and it ranks as the seventh leading cause of death and one of the major reasons for disability and dependency among older people globally. Sterile inflammation in neurodegenerative diseases, like Alzheimer’s (AD) and Parkinson’s (PD), is fueled by endogenous danger signals such as misfolded proteins, oxidative stress, and neuronal cell death [136]. This chronic inflammatory response involves the activation of the microglia and astrocytes and the release of pro-inflammatory cytokines and chemokines in the CNS [137, 138]. Several types of cell death mechanisms have been identified in the pathogenesis of neurodegenerative diseases including apoptosis, necroptosis, pyroptosis, ferroptosis and a special form of neuronal cell death known as excitotoxicity [139] (Table 3). Excitotoxicity is triggered by excessive stimulation of the N-methyl-D-aspartate receptor (NMDA) subtype of glutamate receptors leading to the loss of ATP, causing the general depolarization of neurons, thus the cytosolic Ca^2+^ is sequestered resulting in ER stress and/or mitochondrial permeability transition [140].

**Table 3:** Cell death and DAMP production in neurodegenerative diseases.

Diseases	Risk factor	Cell death	Cell death KO mice	DAMP	DAMP KO mice
**Neurodegenerative diseases**	Smoking [280] Hypertension [281] Obesity [282] Hypercholesterolemia [283] Alcohol [284] Air pollution [285]	Apoptosis [141] Necroptosis [142] Pyroptosis [144] Ferroptosis [146] Excitotoxicity [140]	Delayed neuronal cell death in traumatic brain injury in Bax, RIPK1- or RIPK3-deficient mice [286], [143]. Ablation of NLRP3 and GSDMD exerts neuroprotective effects after traumatic brain injury [149]. Improved pathogenesis of AD in NLRP3 or caspase-1 KO mice [147]. PARP-1 deletion prevents cognitive dysfunction in AD [148]. CypD depletion attenuated synaptic degeneration in an AD mouse model [150].	astrocytes: ATP, HSP70, uric acid S100 neurons: ATP, HSP70, uric acid, mtDNA formylated peptides, HMGB1, PRXs [152] oligodendrocytes and glia cells: HSP70, mtDNA formylated peptides, HMGB1, PRXs [152] Chromogranin A [153]	Knockout of S100B decreases plaque load in the cortical region in AD mouse model [287].

In addition to apoptosis which was demonstrated to be a contributing cell death pathway in neurodegeneration [141], elevated levels of various markers of necroptosis were also detected in the postmortem brain tissues of AD patients. Mechanistically, TNFα production by activated microglia induces necroptosis in astrocytes [142]. Studies on knockout mice mechanistically confirm the role of various cell death processes in neurodegenerative diseases. Data observed in neuron-specific RIPK1- or RIPK3-deficient mice confirmed that necroptosis contributed to delayed neuronal cell death in traumatic brain injury [143].

Furthermore, the levels of key components of pyroptosis, NLRP3, caspase-1, GSDMD, IL-1β and IL-18 were also increased in PBMCs of AD patients [144], and GSDME is found to be highly expressed in the brain tissue and neurons of patients with neurodegenerative diseases executing its role in the pyroptotic pathway [145]. Cortical iron has also been shown to be elevated in AD possibly inducing a ferroptosis-related inflammatory response [146].

Knockdown of NLRP3 or caspase-1 reduced neuronal death and reversed cognitive impairments [147], whereas PARP-1 -/- mice were observed to prevent synaptic damage, cognitive dysfunction, and microglial activation in an AD mouse model referring to the significant role of pyroptosis and parthanathos in the pathogenesis of AD [148, 149]. Accordingly, GSDMD depletion inhibited the expression and release of IL-1β and TNF-α while promoting those of anti-inflammatory cytokines (IL-10 and TGF-β1) in neurodegenerative pathologies [149]. MPT-related cell death mechanisms are also implicated in various neurological diseases based on CyPD knockout studies [150].

Various danger-associated molecules, including HMGB1, ATP, and S100B, participate in neuroinflammation and activate cerebral myeloid cells and other brain cells to produce inflammatory factors. Soluble amyloid β (Aβ) is considered an inducible DAMP molecule, the secretion of which leads to inflammatory cytokine production [151]. HMGB1 seems to colocalize with A*β* in senile plaque that is associated with activated microglia, enhancing A*β* neurotoxicity and inhibiting microglial clearance. Moreover, various cells have been identified as sources of DAMP secretion in neurodegeneration, such as astrocytes (ATP, HSP70, uric acid and S100B), injured neurons (ATP, HSP70, uric acid, mtDNA, formylated peptides, HMGB1, PRXs [152], oligodendrocytes (HSP70) and glia cells (HSP70, mtDNA, formylated peptides, HMGB1 [152]. In addition, Chromogranin A released by neuronal stress or demise stimulates the further release of cytotoxins and neurotoxins by microglia acting as DAMP in AD [153]. Several of these DAMPs (mtDNA, ATP, uric acid) have been implicated in inducing pyroptosis, contributing to the exacerbation of the inflammatory process [154, 155].

## The role of cell death and related DAMP release in diabetes mellitus

Type 2 diabetes (T2D) mellitus is a multifactorial disease with increasing incidence worldwide. The β-cell apoptosis reduces the β-cell mass [156], but different kinds of regulated non-apoptotic cell deaths also contribute to its pathogenesis (Table 4) [157].

**Table 4:** Cell death and DAMP production in type2 diabetes.

	Risk factor	Cell death	Cell death KO mice	DAMP	DAMP KO mice
**T2D**	Smoking [288] Hypertension [289] Obesity [290] Hypercholesterolemia [291] Alcohol [292] Air pollution [28]	Apoptosis [293] Necroptosis [294] Pyroptosis [295] Ferroptosis [157]	Caspase-8 is essential for beta-cell apoptosis in type 1 and type 2 diabetes [161]. MLKL KO mice reveal MLKL as a regulator of insulin sensitivity [65]. Both Nlrp3 and Caspase-1 deficiencies improve glucose tolerance and insulin sensitivity [162], [67]. Sterile inflammation induced by diabetic nephropathy is blunted in both Caspase-11-/- and GSDMD-/- mice [296]. The specific knockdown of Nrf2 increased the sensitivity of cells to ferroptosis in high glucose conditions [297]. Mice lacking CypD were protected from high-fat diet-induced glucose intolerance [163].	mtDNA [165] HMGB1 [298] glucose IAPP palmitate ceramide ECs AGE S100 proteins [164] ROS [299] Uric acid [167]	Decreased glucose levels and increased glucose tolerance was shown in HMGB1 KO mice [166].

Necroptosis plays a role in a form of diabetes named maturity-onset diabetes of the young (MODY) which is an autosomal dominant disease with incomplete penetrance. Interestingly, a very rare heterozygous damaging mutation in MLKL was found exclusively in patients with diabetes [158].

Recent studies have demonstrated that activation of NLRP3 inflammasome contributes to insulin resistance and β-cell death in T2D. Activation of NLRP3 inflammasome has also been observed in macrophages under inflammatory conditions. For instance, in conditions of hypoxia and high glucose levels, the saturated fatty acid palmitate can trigger NLRP3 inflammasome/caspase-1-mediated pyroptosis and consequently IL-1β, and IL-18 production. The loss of β-cells is not the only factor that causes complications; IL-1β production also plays a critical role in chronic inflammation in T2D as it interferes with insulin signaling, resulting in impaired glucose tolerance and reduced insulin sensitivity [159].

Several studies have shown that pancreatic β-cells are predisposed to ferroptosis due to low levels of antioxidant enzymes, such as superoxide dismutase, GPx4, and catalase. Furthermore, Fe^2+^ in the labile iron pool promotes ROS synthesis via the Fenton reaction in β-cells. In addition, ferroptosis in the liver, fat, and muscles also causes insulin resistance [160].

The role of different cell death pathways in T2D has also been intensively investigated in knock-out mouse models, mice lacking Caspase-8 in β-cells were protected from in vivo models of type 1 and type 2 diabetes, but with aging, these mice gradually developed hyperglycemia and a concomitant decline in β-cells mass, confirming that several different cell death pathways may play a role in T2D [161]. Whole-body deficiency of MLKL [65], NLRP3 [67, 162], or CyPD [163] also prevented obesity-induced, or high-fat diet (HFD)-induced insulin resistance and glucose intolerance.

Increased levels of various DAMPs, including RAGE ligands such as advanced glycation end products (AGE), HMGB1, S100 proteins, mtDNA, uric acid, and ROS, have been reported in both diabetic patients and animal models. Either AGE, HMGB1, S100, or mtDNA induce AIM2 or NLRP3 inflammasome activation which plays a critical role in the pathogenesis of type 2 diabetes [164, 165]. Elevated levels of these DAMPs regulate the secretion of the proinflammatory cytokines IL-1β and IL-18, leading to chronic inflammation. Accordingly, a reduction in glucose levels and increased glucose tolerance has been observed in HMGB1 knockout mice [166]. Elevated uric acid levels also increase the secretion of IL-6 and TNFα, in addition to the production of IL-1β. High blood glucose, glycemic variability, hypoglycemia, and uric acid are all related to increased ROS production, which leads to the development of insulin resistance and impaired insulin secretion [164, 167].

## The role of cell death and related DAMP release in liver diseases

Hepatocyte cell death is a critical event in the progression of liver disease due to resultant inflammation leading to fibrosis. Apoptosis, necroptosis, pyroptosis, and ferroptosis have all been investigated in the pathogenesis of various liver diseases, such as alcoholic liver disease (ALD), non-alcoholic fatty liver disease, steatohepatitis (NAFLD/NASH), acetaminophen-induced hepatotoxicity, autoimmune hepatitis and cholestatic liver disease [168]. In this chapter, we review the two most prevalent types of liver conditions, ALD and NASH/NAFLD (Table 5).

**Table 5:** Cell death and DAMP production in liver diseases.

Diseases	Risk factor	Cell death	Cell death KO mice	DAMP	DAMP KO mice
**Alcoholic Liver Disease (ALD)**	Smoking [300] Hypertension [301] Obesity [302] Hypercholesterolemia [303] Alcohol [304] Air pollution [305]	Apoptosis [170] Necroptosis [116] Pyroptosis [180] Ferroptosis [121]	Bid-/- mice were protected from EtOH-induced apoptosis, but Bid not contribute to EtOH-induced steatosis and hepatocyte injury [170]. Alcohol-treated RIPK3 KO mice had less steatosis, inflammation, and liver damage [116], [171]. Deficiency of NLRP3, Caspase-1, ASC, caspase-11 and IL-1R1, prevented alcohol-induced inflammation and liver damage [180], [119], [173].	Glutamate HMGB1 ATP miRNA Mitochondrial dsRNA Mitochondrial DNA Nuclear DNA EV ligands Lipids [122]	HMGB1 ablation in hepatocytes protected mice from alcohol-induced liver damage [179].
**Nonalcoholic Fatty Liver Disease NASH/NAFLD**	Smoking [306] Hypertension [307] Obesity [308] Hypercholesterolemia [309] Alcohol [310] Air pollution [311]	Apoptosis [312] Necroptosis [313] Pyroptosis [175] Ferroptosis [176] Mitochondrial Permeability Transition [314]	Reduced inflammation was shown in Caspase-3 and Caspase-8 deficient mice [312], [315]. Steatohepatitis and NAFLD were reduced in high-fat diet-fed MLKL-KO mice [313]. In GSDMD deficient mice steatohepatitis and the level of inflammatory cytokines was reduced [175]. CypD deficiency reduced high-fat diet-induced hepatic steatosis [314].	ATP Uric acid Fatty acids Cholesterol HMGB1 [316]	

ALD is associated with a complex pathogenesis involving sterile inflammation and is the second most common cause of annual human mortality [169]. Alcohol metabolism via alcohol dehydrogenase, aldehyde dehydrogenase, cytochrome P450 2E1, and catalase increases ROS production, which triggers mitochondrial damage and hepatocyte cell death. However, alcohol-induced hepatocyte apoptosis does not account for the inflammatory cytokine production observed in mice after chronic alcohol feeding [170]. In contrast to apoptosis, inhibition of necroptosis or pyroptosis was shown to reduce alcohol-induced inflammation. RIPK3 knockout mice had attenuated levels of proinflammatory cytokines and chemokines, and decreased plasma aspartate aminotransferase (AST) and alanine aminotransferase (ALT) activities during ethanol-induced liver damage or Gao-binge alcohol treatment [116, 171]. However, the role of MLKL in ALD remains questionable [172]. Biopsy samples from ALD showed increased RIPK3 expression, but not RIPK1 expression [116]. Also, the deficiency of NLRP3, Caspase-1, ASC, IL-1R1, and Caspase-11 prevents alcohol-induced liver inflammation and liver damage [119, 173].

It is currently unknown how ferroptosis plays a role in the pathogenic mechanism of alcoholic steatohepatitis. Nevertheless, long-term ethanol feeding increased the expression of ferroptosis-related genes, lipid peroxidation, and the amount of labile iron. Ethanol can also sensitize the liver to ferroptosis by depleting cysteine and suppressing the xCT glutamate antiporter [121, 174].

NAFLD is the most common cause of liver disease in the U.S. NASH occurs in 20% of NAFLD, in which hepatocyte cell death causes inflammation and fibrosis [168]. While apoptosis is undoubtedly important in the development of NASH [168], the role of necroptosis in this disease is highly controversial depending on the model used and the molecules knocked down [172]. While MLKL KO mice were protected from steatohepatitis in various models, symptoms could be exacerbated in RIPK3-/- mice. Presumably, RIPK3 deficiency exacerbated apoptosis in various models [172].

Furthermore, pyroptosis in hepatocytes and macrophages is also involved in the development of liver fibrosis in NAFLD, as the pro-inflammatory cytokines released during pyroptosis are key molecules for NAFLD development [168]. In GSDMD KO mice fed with HFD, liver histology was significantly improved, steatohepatitis and the level of inflammatory cytokines were reduced, and elevated levels of the cleaved GSDMD-N fragment were detected in liver biopsy samples from patients with NASH [175].

The liver plays a significant role in iron metabolism and hepatic lipogenesis, and abnormal lipid accumulation promotes hepatic steatosis in NASH/NAFLD, accordingly, ferroptosis also takes on an important part in the disease’s pathogenesis [176]. Iron overload is a key characteristic of NAFLD, and elevated serum ferritin levels have been observed in patients with this disease [177]. Steatosis and inflammation were significantly improved using ferroptosis inhibitors in mice exposed to a methionine- and choline-deficient diet, a classical dietary model of NASH [178].

Hepatocytes release HMGB1 in a dose- and time-dependent manner in response to ethanol treatment in vitro, and an increase in HMGB1 expression was detected in liver biopsies from patients with ALD. HMGB1 seems to be a dominant DAMP in ALD, since HMGB1 ablation in hepatocytes protected mice from alcohol-induced liver injury [179]. Ethanol-damaged hepatocytes release uric acid and ATP, activating the inflammasome and IL-1β-related inflammation. This creates a pyroptosis-mediated cycle that maintains inflammation in ALD. Alcohol metabolism in liver cells further increases the production of ROS, which results in mitochondrial dysfunction [180, 181].

## Clinical opportunities for regulating cell death in fatal diseases

Understanding the interplay between sterile inflammation, cell death and DAMP release offers potential targets for therapeutic intervention to mitigate disease progression. Several diseases are linked to risk factors, regulated necrosis, or DAMP production, but only a small number of approved drugs have been developed to inhibit cell death or DAMP action.

Emricasan (approved for the treatment of non-alcoholic steatohepatitis cirrhosis) as an apoptosis inhibitor, Rilonacept (used for the treatment of cryopyrin-associated periodic syndromes), Canakinumab (for the treatment of systemic juvenile idiopathic arthritis, active Still’s disease) as inhibitors or antibodies neutralizing IL-1β, or Anakinra (used to treat rheumatoid arthritis, cryopyrin-associated periodic syndromes, familial Mediterranean fever, and Still’s disease) as an IL-1R antagonist are currently used. These drugs are also being studied in clinical trials focusing on cardiovascular diseases [182], neurodegenerative diseases [183], diabetes [184] and ALD [185]. Four PARP inhibitors [186] niraparib, olaparib, rucaparib, and talazoparib, have been approved for the treatment of various cancers by inhibiting DNA damage repair. Since these drugs could potentially be used to block cell death as well, strategies to block parthanatos offer promising therapeutic approaches for the treatment of parthanatos-related disorders such as neurodegenerative diseases. For example, veliparib, rucaparib, and talazoparib were shown to prevent the α-synuclein preformed fibrils-mediated cell death in mice PD models. In ALS veliparib and olaparib can inhibit TAR DNA-binding protein 43-associated neuronal death. A PARP-1 inhibitor, PJ34, inhibits Aβ-induced neuronal death in AD models, and another PARP-1 inhibitor, INO-1001, has shown neuroprotective effects in a mouse model of human Huntington’s disease [187].

Efforts to inhibit necroptosis have so far focused on RIPK1, but the development of probes targeting RIPK3 and MLKL would also be essential. Off-target activity, species specificity, and interference with non-necroptosis functions of RIPK1 and RIPK3 pose challenges to the development of a successful necroptosis inhibitor [188].

In recent years, research into small-molecule drugs that inhibit pyroptosis has received more and more attention. For example, INF4E, a newly synthesized small-molecule inhibitor of NLRP3, significantly inhibits the release of LDH characteristic of necrosis, moderates the development of NLRP3-dependent inflammation, and reduces infarct size. Disulfiram, a drug used to treat alcohol dependence, has been shown to effectively inhibit GSDMD pore formation in human and mouse cells, thereby inhibiting pyroptosis [189].

Considerable progress has been made in developing pharmacological agonists and antagonists for the treatment of these ferroptosis-related conditions. As an antioxidant, (*N*-acetylcysteine NAC) has been shown to inhibit ferroptosis by targeting cysteine metabolism. It is clinically approved to treat acetaminophen toxicity overdose and NAC has also been clinically shown to improve neurodegeneration-related symptoms by increasing cysteine levels and facilitating the synthesis of γ-glutamyl-cysteine and GSH. A modified form of NAC with increased membrane permeability, NACA, is being tested to mitigate the side effects of NAC. To date, NACA has been shown to have antioxidant effects in several preclinical models but is still awaiting approval for clinical use. Both the ALOX15-specific inhibitor ML351 and Baicalein, a specific ALOX12 antagonist, protect against I/R-induced cardiac injury in mice. Polyunsaturated fatty acids deuterated at the bis-allyl position have also been shown to inhibit ferroptosis in animal models of PD and Friedreich’s ataxia [190]. In addition, copper-diacetyl-bis(N4-methylthiosemicarbazone), a candidate drug for treating ALS and PD, suppresses ferroptosis via its RTA activity [191].

Regulation of NINJ1, a common point of various necrotic cell death processes, is a new therapeutic approach to downregulate inflammation. The anti-NINJ1 neutralizing monoclonal antibody attenuated inflammation induced by liver damage or ischemia-reperfusion injury and also reduced gout flare [192, 193].

Prophylactic anti-HMGB1 therapy constitutes a promising new tool to reduce HMGB1-dependent inflammation/damage and improve patient outcomes. For example, pretreatment with HMGB1 antibody protects against secondary liver damage and ameliorates renal dysfunction due to ischemia-reperfusion in vivo. Intraperitoneal injection of a monoclonal anti-HMGB1 has been shown to attenuate serum IL-17 elevation and reduce the demyelination associated with experimental autoimmune encephalomyelitis [194].

For deeper understanding, current improvements targeting necroptosis [188], pyroptosis [189], ferroptosis [191], parthanatos [187], or HMGB1 [194] are included in the indicated review articles.

Similar cell death or DAMP secretion processes are behind various diseases, which suggests that drugs that are effective in certain diseases should also be tested in other diseases with intensive cross-examination. This is confirmed by the fact that several drugs currently preferred in the treatment of human cardiovascular diseases, neurodegenerative disorders and diabetes can also regulate cell death in other indications according to mouse models (Table 6). A more precise exploration of the regulatory functions of cell death would expand the applicability in humans of these agents to other diseases as well.

**Table 6:** Approved drugs used in human cardiovascular and neurodegenerative diseases, type 2 diabetes, ALD and NAFLD that regulate cell death in various tissues based on in vivo mouse studies.

Diseases in which drugs: control cell death, according to in vivo studies in mice	Diseases in which drugs: are approved for human use			
	Cardiovascular diseases	Neurodegenerative diseases	T2D	ALD/NAFLD
**Cardiovascular diseases**	Ezetimibe **A** [317] Ticagrelor **P** [318] Inclisiran **P** [319] Colchicine **P** [320] Atorvastatin **P** [321]	Edaravone **A** [322, 323] combination of Benidipine and Edaravone **A** [324]	Metformin **N** [325] **P** [326] Pioglitazone t **P** [327] Dapagliflozin **F** [328]	**-**
**Neurodegenerative diseases**		Rasagiline and Selegiline **A** [329] Edaravone **A** [330] Memantine **A** [331], [332] Y-2 **N** [333] L-DOPA **P** [334]	Dapagliflozin **A** [335] Sitagliptin **A** [336] Pioglitazone **P** [337] Thiazolidinediones **F** [338]	**-**
**T2D**	Sacubitril/valsartan **A** [339]		SGLT2 inhibitor with Linagliptin **A** [340] Linagliptin **N** [341] Empagliflozin **P** [342]	**-**
**ALD/NAFLD**	Pentoxifylline and Kaempferol **A** [343]	Geniposide **A** [344]	Empagliflozin **A** [345] Tirzepatide **A** [346]	**-**

*A* apoptosis, *N* necroptosis, *P* pyroptosis, *F* ferroptosis.

Several other approved drugs have been found to influence cell death processes by either activating or inhibiting cell death mechanisms, suggesting exploring these drugs for use in treating sterile inflammation-related diseases. Here we refer to the studies related to necroptosis [195], pyroptosis [196, 197] and ferroptosis [191].

## Conclusions

Sterile inflammation takes place in several steps, different stimuli induce regulated necrosis. Innate immune cells, mainly macrophages and DCs, respond to the resultant DAMPs release by producing inflammatory mediators. Consequently, some mediators like TNFα and interferons, certain DAMPs can trigger further cell death, establishing a feedback loop leading to chronic reactions, unless pro-resolving pathways are dominante. The tissue destruction characteristic of various diseases can be caused by several mechanisms, even simultaneously. Cell death can be induced [1] directly by risk factors, [2] due to inflammatory effector mechanisms triggered by DAMP release during necrotic cell death and [3] by exacerbation of inflammation through necrotic cell death induced by secreted DAMPs. It is not entirely known to what extent these effects are involved in the development or progression of each disease, and which effects cause inflammation to become a chronic process. Current anti-inflammatory treatments do not address the root cause of either the cell death process or the dysregulated release of DAMPs. A deeper understanding of cell death and DAMPs’ role in the disease pathogenesis could enable causal, rather than merely symptomatic therapy.

## Data Availability

All data generated or analyzed during this study are included in this published article.
